# Fuzzy‑set qualitative comparative analysis and fuzzy cognitive maps: Exploring pregnancy outcomes and maternal depression

**DOI:** 10.3892/mi.2025.229

**Published:** 2025-03-27

**Authors:** Antigoni Sarantaki, Anastasia Nomikou, Katerina Tzimourta, Eirini Orovou, Kleanthi Gourounti, Stavroula Barbounaki

**Affiliations:** 1 Midwifery Department, Faculty of Health and Care Sciences, University of West Attica, 12243 Athens, Greece; 2 Biomedical Technology and Digital Health Laboratory, Department of Electrical and Computer Engineering, Faculty of Engineering, University of Western Macedonia, 50100 Kozani, Greece; 3 Midwifery Department, University of Western Macedonia, 50100 Kozani, Greece; 4 Merchant Marine Academy of Aspropyrgos, 19300 Athens, Greece

**Keywords:** antenatal attachment, antenatal depression, decision-making, pregnancy outcome, fuzzy-set qualitative comparative analysis, fuzzy cognitive map

## Abstract

The maternal antenatal attachment scale (MAAS), the pregnancy outcome questionnaire (POQ) and the Centre for Epidemiologic Studies Depression Scale (CESD), among other approaches, have been developed to address pregnancy-related psychological issues. However, the need to develop and validate effective scales to screen the complex experiences of pregnant women continues to be extensively discussed in the literature. The aim of the present study was to build and validate fuzzy models that represent the necessary and sufficient causal combinations that lead to higher levels of anxiety regarding pregnancy outcomes, maternal prenatal attachment to the unborn child and depressive symptoms, respectively. For this purpose, measurements from the MAAS, POQ and CESD scales, along with demographic data, were collected from 135 pregnant women, including cases of natural conception (NC) and assisted reproduction (ART) births. Fuzzy-set qualitative comparative analysis (FSQCA) was employed to produce sets of causal combinations, which were validated against their consistency and coverage. These combinations were then used to develop and validate fuzzy cognitive maps (FCMs) to model the fluctuations in the status of pregnant women. To the best of our knowledge, the present study is the first to utilize FSQCA or FCM to address this issue. The results indicated that the POQ was the distinguishing factor between NC and ART that led to higher MAAS levels. Marital status (MS) and state anxiety were found to lead to higher POQ levels for pregnancies derived from NC. For pregnancies following ART, the factors to consider include income, week of pregnancy, MS, MAAS intensity and trait anxiety. POQ was found to lead to higher levels of CESD for ART pregnancies, while NC, MS and state anxiety are also prerequisites. On the whole, the present study demonstrates that the proposed FSQCA- and FCM-based approach enables obstetricians and midwives to incorporate their expertise in evaluating cases on an individual basis, while also providing a framework for creating intelligent systems to support healthcare policy decisions.

## Introduction

Pregnancy is one of the most complex experiences in the life of a woman. It is a transitional period during which a woman faces unprecedented and pivotal changes in body image, self-perception, daily life and relationships. It is argued that in all pregnancies, there is a level of uncertainty for the woman, for her ability to carry the pregnancy to term, a degree of stress about the forthcoming childbirth, and concerns about the health and normal development of the fetus (1-3). This process can be demanding for some women, as their self-perception evolves to embrace the new role of motherhood (4).

Achieving this change requires accepting the pregnancy, identifying with the role of the mother, reorganizing the relationship with their own mother and partner, developing a bond with their unborn child and preparing for childbirth (5). The transition to this new role does not occur naturally for the majority of women, as it causes hormonal and neurochemical adjustments in both males and females, as well as psychological changes regarding individual identity (6,7). Extensive research into depression and pregnancy anxiety, suggests that emotional disturbances occur more frequently during this time than was previously considered (8-14).

Despite the considerable research on pregnancy-related anxiety, there is criticism regarding the predictive validity of the existing scales, highlighting the need to develop effective screening instruments (10,15). In the present study, it was hypothesized that fuzzy logic approaches, specifically fuzzy-set qualitative comparative analysis (FSQCA) and fuzzy cognitive maps (FCMs), capture the nuanced interplay between psychosocial and demographic factors, thus providing more personalized assessments. It was hypothesized that symptoms of anxiety and depression during pregnancy are influenced by unique combinations of demographic and psychological factors, which can be modeled using the FSQCA. Fuzzy models provide a more holistic understanding of maternal-fetal attachment compared to traditional statistical approaches, considering predictor interdependencies. Socioeconomic status, marital satisfaction, and trait anxiety are the key determinants of pregnancy-related psychological outcomes.

Although the potential of fuzzy logic systems has been highlighted in a number of previous medical studies (16-27), its application in obstetrics and midwifery is completely overlooked. In medical assessment and decision-making, intuition, judgment and intangible knowledge acquired by experts through years of experience are often difficult to model and analyze. Currently, statistical methods, particularly, regression techniques, are popular in medical domains; however, regression fails to test the multiple interactions among independent factors (28). Regression estimates the magnitude and direction of the effect of a variable, net of other variables included in the model (29).

By contrast, the FSQCA focuses on identifying the necessary and sufficient conditions leading to a given outcome (29). Fuzzy logic provides the necessary methods to process uncertainty, imprecision and partial truth, enabling the capture and analysis of intuition and subjectivity and the development of intelligent systems and algorithms that more closely resemble human reasoning than other approaches. Thus, the need for further research in applying fuzzy logic to medical issues has been suggested by a number of researchers (22,26,27).

Given the complexities of maternal psychological well-being and the limitations of traditional statistical methods in capturing non-linear associations, the present study employed an innovative approach by integrating FSQCA and FCM. By identifying necessary and sufficient factor combinations, FSQCA can provide a structured framework for understanding the diverse experiences of pregnant women, while FCM may enable the dynamic modeling of these influences over time.

To meet these objectives, the present study implemented a survey-based analysis, obtaining data from a strategically selected cohort of pregnant women in both public and private healthcare settings. The present study aimed to illustrate the applicability of fuzzy logic by utilizing FSQCA to produce the necessary and sufficient conditions that independently lead to higher levels of anxiety about pregnancy outcomes, maternal prenatal attachment to the unborn child and depressive symptoms, specifically targeting primiparas who had surpassed 24 weeks of gestation with a single fetus, following either spontaneous conception or assisted reproduction. The causal combinations are subsequently used to develop FCM, which, after validation, are utilized to assess fluctuations in the status of pregnant women status concerning the output variables.

## Subjects and methods

### Study subjects

A cross-sectional questionnaire-based methodology was employed, targeting a purposive sample of first-time pregnant women after the 24th week of singleton pregnancies. The recruitment process was conducted in several private and public prenatal care facilities from October, 2023 to July, 2024, when the study was introduced to eligible individuals by maternity care providers during routine antenatal check-ups.

The eligibility criteria for the present study were designed to ensure a focused and relevant participant pool. Primiparity was a key inclusion criterion, allowing researchers to examine the experiences of women navigating pregnancy and childbirth for the first time. This focus on primiparas helps control for potential confounding factors from previous pregnancies. The present study included only singleton pregnancies beyond the 24th gestational week, ensuring a more homogeneous sample and excluding early pregnancy complications.

Exclusion criteria were established to refine the study population and maintain internal validity. Multiple pregnancies were excluded due to their unique challenges, which could introduce variables that may skew the results. Pregnancies with known fetal anomalies were not included, as these cases often require specialized care and may involve different factors compared to uncomplicated pregnancies.

To ensure the accuracy of data collection and interpretation, particularly in questionnaires and other communicative aspects, the present study excluded individuals who lacked proficiency in Greek, which was the language employed in the current research. The established criteria facilitated the researchers' development of a more homogeneous study population, thereby enhancing the validity of findings within the context of primigravid, singleton pregnancies.

Potential participants were provided with comprehensive information regarding the objectives of the study, study protocols and data confidentiality. Informed consent was obtained from all participants who agreed to partake in the study, ensuring they understood their involvement and rights. This procedure facilitated the collection of a diverse set of data pertinent to the study objectives, focusing on the necessary conditions that independently lead to various pregnancy-related outcomes. Data collection involved the administration of the maternal antenatal attachment scale (MAAS), pregnancy outcome questionnaire (POQ) and the Center for Epidemiologic Studies Depression Scale (CESD), along with demographic data. A cohort of 135 subjects meeting the inclusion criteria was recruited over a 6-month period.

The research design and data protection measures were assessed and approved by the Ethics Committee of the University of West Attica (registration approval code 95812/10-10-2023) to guarantee adherence to ethical standards. Data protection measures included anonymizing participant identities and storing information on secure password-protected servers, with access limited to authorized researchers only. Data confidentiality was maintained throughout the study period. Moreover, a data use agreement was established to assure participants that their information would not be shared with third parties and would only be used for the specified research purposes.

No control group was included due to logistical constraints, as the methodologies employed, particularly FSQCA and FCMs, do not require a traditional control group to identify meaningful patterns. The present study explored the application of fuzzy logic in medical contexts by implementing FSQCA and FCM techniques. The investigation gathered information from expectant mothers to create fuzzy models that enable individualized evaluations. The following sections describe the steps of the proposed approach.

### Step 1: Collection of data regarding female demographics and MAAS, POQ and CESD

A thorough analysis of the relevant literature was performed to select a comprehensive set of factors that affect the output variables i.e., measurements of the MAAS, POQ and CESD scales before birth, both in the case of births derived from natural conception (NC) or assisted reproduction (ART) (21,30). The present study analyzed data collected from a sample of 135 expectant individuals. The researchers prioritized data integrity by excluding any incomplete responses from the analysis. Before commencing data collection, the objectives and parameters of the study were fully communicated to all participants, who subsequently provided their informed consent. The average age of the study participants was 33.02 years of age with a standard deviation of 5.16; thus, the minimum and maximum age was 22 and 50 years, respectively. The majority of the participants (115 women) conceived naturally and 20 women achieved pregnancy following ART.

### Step 2: Fuzzification of selected data

The selected data are defined as triangular fuzzy number (TFN). The membership function *f_A_*(*χ*) of TFN *Ã(a, m, b)*, where a, m, b are real numbers, can be calculated according to the following equation [1].


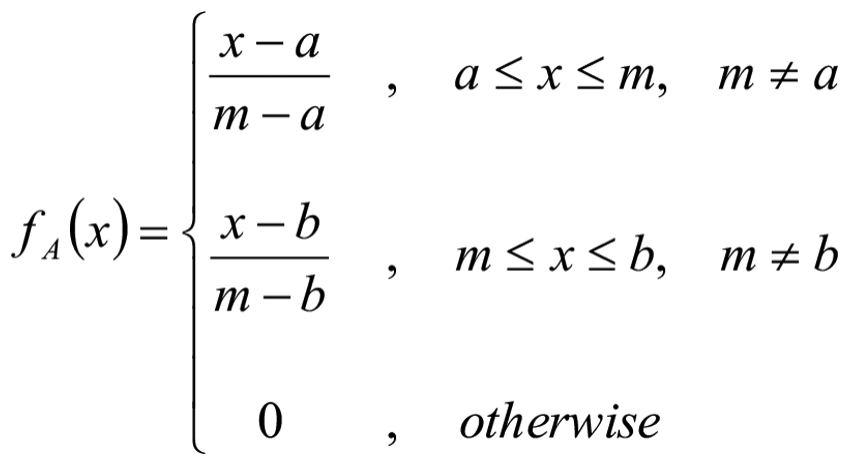
 [1]

This equation represents the membership function of a TFN, where \(a \), \(m \) and \(b \) define the fuzzy set. The TFN for each concept included in the present study is defined by considering the data intervals (minimum and maximum values) of each scale considered in the study and consulting the opinions of experts. The TFN used in the present study is presented in Table I. The present study used TFNs as they work best to consolidate fragmented expert opinions (31) and are closer to human thinking (32).

### Step 3: Application of FSQCA and calculate consistency and coverage of causal combinations

There were 9 cases considered in the present. The truth table of all possible permutations of input variables is produced in each case scenario. In general, if there are (k) input variables, then the size of the truth table is 2^k^. The causal combinations' membership degrees are calculated according to the fuzzy logic operations rules (equations 2-4 below). The proposed approach was tested for its validity by calculating the consistency and coverage of the produced causal combinations which are calculated by using the formulas (equations 5 and 6) adapted from the study by Korjani and Mendel (33). According to FSQCA, the optimal causal combinations should exhibit consistency and coverage as high as possible. However, the higher the consistency is getting the lower the coverage it becomes.

The output and the input variables as well as the sample considered in each case are presented in Table II. The present study adopted a consistency threshold value of 0.8 from the study by Skarmeas *et al* (34) and then identified the causal combinations with coverage as high as possible. Usually, acceptable coverage rates (35) are within the interval (0.25 and 0.65).

According to FSQCA, the optimal causal combinations should exhibit both high consistency and coverage. However, there is an inherent trade-off between these two metrics: As consistency increases, coverage tends to decrease. To ensure robust yet interpretable results, the present study adopted a consistency threshold of 0.8, as suggested by Skarmeas *et al* (34). This threshold is widely recognized in FSQCA literature as an appropriate benchmark for distinguishing meaningful causal patterns from random associations.

Furthermore, the acceptable range for coverage rates, typically between 0.25 and 0.65(35), is based on previous empirical studies and methodological guidelines in FSQCA research. Coverage quantifies the extent to which a causal combination accounts for the outcome, and values within this range strike a balance between capturing relevant explanatory power and avoiding overly broad, less informative configurations. By selecting a consistency threshold of 0.8 and prioritizing the highest possible coverage within the acceptable range, this study ensures methodological rigor while maintaining meaningful and interpretable causal pathways.

### Step 4: Producing the set of causal combinations

The causal combinations that satisfy the consistency and coverage thresholds are selected to form the set of alternative causal paths. The Karnaugh Map (36) was utilized to simplify the set of causal combinations thus producing parsimonious models.

### Step 5: Development and validation of a FCM for each case

The present study developed a FCM for each parsimonious causal combination produced by FSQCA. In total, seven expert obstetricians agreed to participate in the study and expressed their beliefs regarding the strength of each causal relationship in the FCM. The geometric means were then applied to determine the aggregated strength for each causal relationship. Concerning the validation of the FCM model and the assessment of its predictive value, the t-test was applied to examine whether a statistically significant difference exists between the model estimations and the actual data in the sample.

*FSQCA.* The FSQCA is particularly critical for investigating intertwined associations among possible causes, which are represented as fuzzy sets and affect a specified output variable (33-39). According to Elliott (29), the FSQCA exhibits numerous advantages over traditional correlational analysis such as regression analysis. More specifically, the FSQCA analysis caters to asymmetrical associations between concepts as opposed to associations calculated from a contingency table. It addresses equifinality in problem-solving, which means multiple solutions may exist for a problem. The FSQCA produces all necessary and sufficient alternative paths to solving a problem and tackles the complexity of a domain problem. In FSQCA, all possible combinations of causal variables that produce an outcome are considered, thus producing the combined effect. Finally, by utilizing fuzzy logic methods such as the FSQCA and FCM research studies can be conducted and empirically assessed even with small sample sizes (33). The steps of FSQCA are as follows:

i) The FSQCA first assumes the truth table of all possible permutations among the multiple factors considered in a study. Each permutation is a possible causal combination.

ii) Subsequently, it calculates the membership degrees for each combination drawing on the fuzzy logic operations theory. Assume two fuzzy sets *Ã* and *B*~ then: The fuzzy union, is defined as: *μ_(A_*_∪_*_B)_* = max*_(__μ__A_,_μ__B)_*, [2]

This formula defines the fuzzy union operation, which determines the degree of membership for the union of two fuzzy sets.

The fuzzy intersection is defined as: *μ_(A_*_∩_*_B)_* = min*_(__μ__A_,_μ__B)_*, [3]

This formula represents the fuzzy intersection operation, which calculates the minimum degree of membership between two fuzzy sets.

The fuzzy complement is calculated as: *μ*_¬_*_A_ =* 1 – *μ_A_* [4]

This formula calculates the complement of a fuzzy set, indicating the degree to which an element does not belong to the set.

iii) Calculate the consistency and the coverage for every causal combination using the formulas [5 and 6] adopted from a previous study (25):


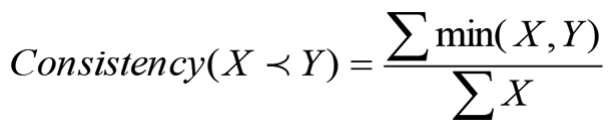
 [5]

This equation is used to determine the consistency of causal combinations in FSQCA, ensuring that selected combinations reliably lead to the expected outcome.


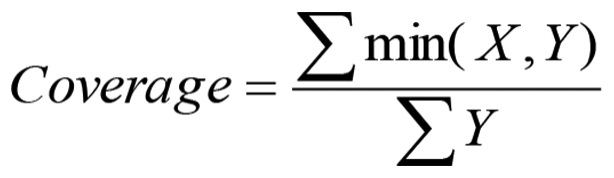
 [6]

This equation calculates the coverage of causal combinations in FSQCA, indicating how much of the outcome variability is explained by a given causal combination.

iv) Select causal combinations with consistency greater than the usual 0,8 threshold and the highest possible coverage (34).

v) Simplify selected causal combinations. The present study applied the Karnaugh Map (36) to produce a more parsimonious model.Ã

### FCMs

An FCM is a graph model that represents the causal relationships among its concepts (40). The strengths of the causal relationships are defined in terms of fuzzy sets, e.g., high, medium, low. FCMs, represented with a matrix, are used to model complex problems, to assess cases and to analyze scenarios and routes of actions. FCMs can be represented by an NxN matrix A=[α_i,j_], where N is the number of the concepts in the FCM and α_i,j_, to represent the strengths between concept (i) and concept (j). The strength of causality for each relationship α_i,j_ take on values from interval [-1, +1]. i) If α_i,j_ >0 then the causal relationship indicates an increase from concept (i) to (j); ii) if α_i,j_=0 then there is no causality between the concepts; iii) if α_i,j_ <0 then the causal relationship indicates decrease from concept (i) to (j). The popular inference rule for estimating the causal propagation in an FCM as previously proposed (41) is as follows:


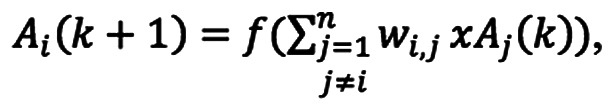
 [7]

This equation defines the inference rule used FCMs, where the activation of a concept at time\ (k+1 \) depends on its previous activation and causal relationships, where A_i_ (k + 1) and A_j_ (k) represent the value of variable A at time k + 1 and k, respectively. Causal effects can be represented with the activation vector (D), which is a 1xN vector. The impact of causal propagation is calculated through repeated multiplications: A x D1=D2, A x D2=D3 and so on, until equilibrium is reached, which is the result of the effect D1. FCM have been widely used in many problems in diagnosis, assessment, etc. (42,43).

## Results

As demonstrated in Τable II, the present study examined 9 cases. Case 1 considers the complete data sample, and its analysis is illustrated in this section. In case 1, causal combinations for assessing MAAS total before birth were examined.

A total of 2^9^=512 causal combinations were examined for the MAAS total. All possible permutations are examined to consider all possible combinations of the status of women. The respective consistency and coverage of the causal combinations were calculated. In Table III, only the causal combinations that exhibited a relatively high consistency (>0.9) and the highest coverage (>0.4) are presented. In each causal combination, the numbers 0 and 1 indicate that the corresponding variable is assumed false or true, respectively. For combination number 8, age=0 indicates a woman of a young age and MS=1 indicates a woman that enjoys a high level of marital satisfaction.

The use of the Karnaugh map (36) produces the following set of cause-effect relationships: IF (not AGE) AND (not POQ) AND (MS) AND (not STATE ANXIETY) AND (not TRAIT ANXIETY) AND (not CESD) OR IF (not EDUCATION) AND (not POQ) AND (MS) AND (not STATE ANXIETY) AND (not TRAIT ANXIETY) AND (not CESD) OR IF (not INCOME) AND (not POQ) AND (MS) AND (not STATE ANXIETY) AND (not TRAIT ANXIETY) AND (not CESD) OR IF (not WEEK of Pregnancy) AND (not POQ) AND (MS) AND (not STATE ANXIETY) AND (not TRAIT ANXIETY) AND (not CESD) THEN <High Level of MAAS before birth>.

The simplification of the cause-effect set of rules returns the (c1) causal combination, which is necessary and sufficient rule to consider in order to estimate if a pregnant woman is expected to exhibit a high Level of MAAS measurement before birth.

IF (not POQ) AND (MS) AND (not STATE ANXIETY) AND (not TRAIT ANXIETY) AND (not CESD) THEN <High Level of MAAS before birth> (c1).

**Figure 1:**
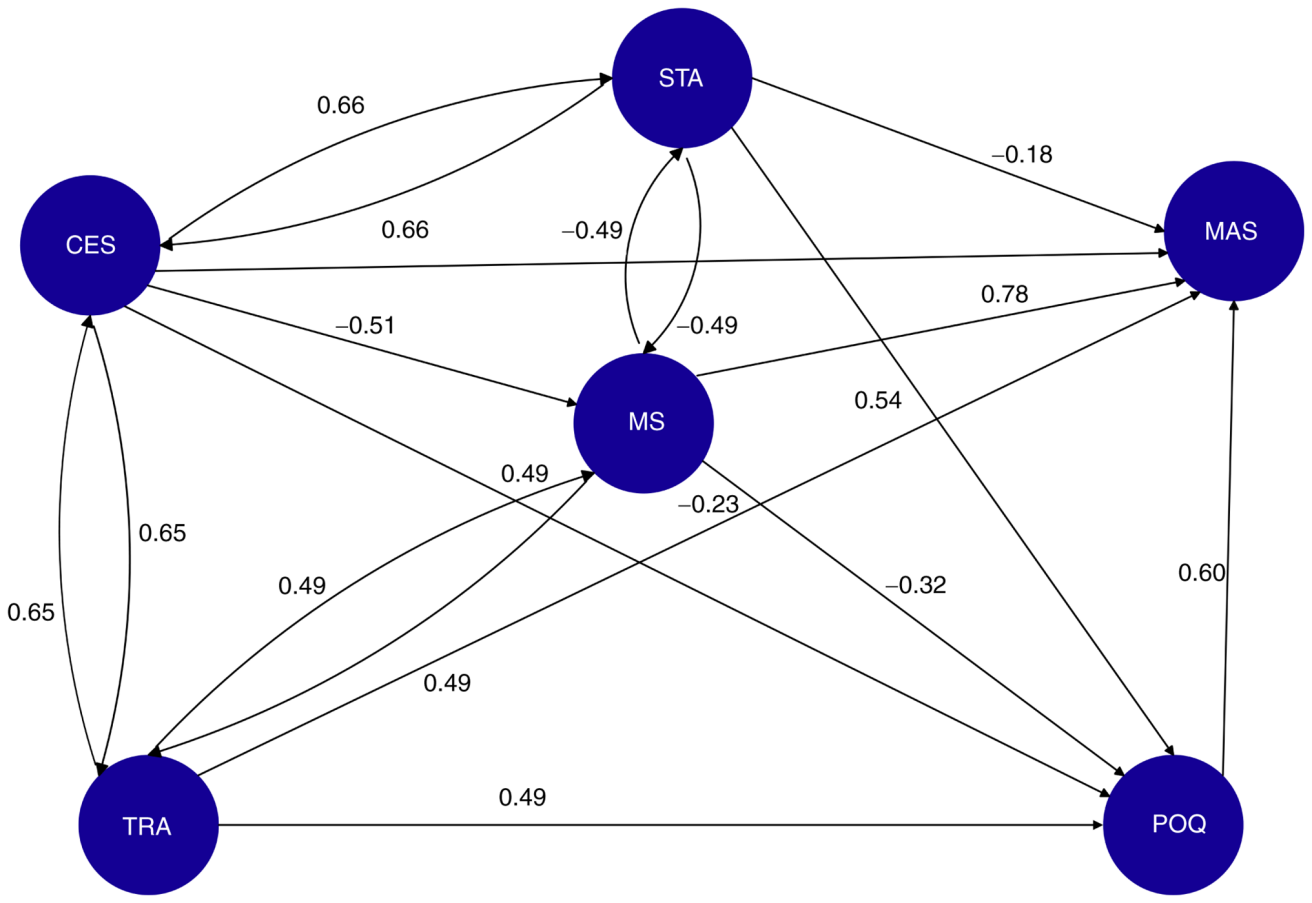
Fuzzy cognitive map illustrates the causal relationships between key factors influencing maternal antenatal attachment (MAAS), pregnancy outcome concerns (POQ), and depressive symptoms (CESD). Nodes represent variables, while directed edges indicate causal influence, with weights derived from expert obstetricians' evaluations. Positive weights signify reinforcing relationships, whereas negative weights indicate inverse relationships. The model predicts how changes in input variables (e.g., marital satisfaction, anxiety, and socioeconomic status) impact maternal psychological well-being during pregnancy. CESD, Centre for Epidemiologic Studies Depression Scale; MAAS, STATE ANXIETY, maternal antenatal attachment scale; POQ, pregnancy outcome questionnaire; MS, TRAIT ANXIETY, marital status, marital satisfaction.

The FCM is illustrated in Fig. 1. The FCM is designed and the weights on its associations are calculated after consulting the group of the seven expert obstetricians. The FCM is used to assess how the changes in the status of a pregnant woman affect the output variables. An activation vector is formed for each pregnant woman case, taking on as values the fuzzified fluctuations of the FCM concepts. The activation vector (MAAS-total-Activation Vector) using woman-1 sample data is presented in Table IV.

**Figure 2:**
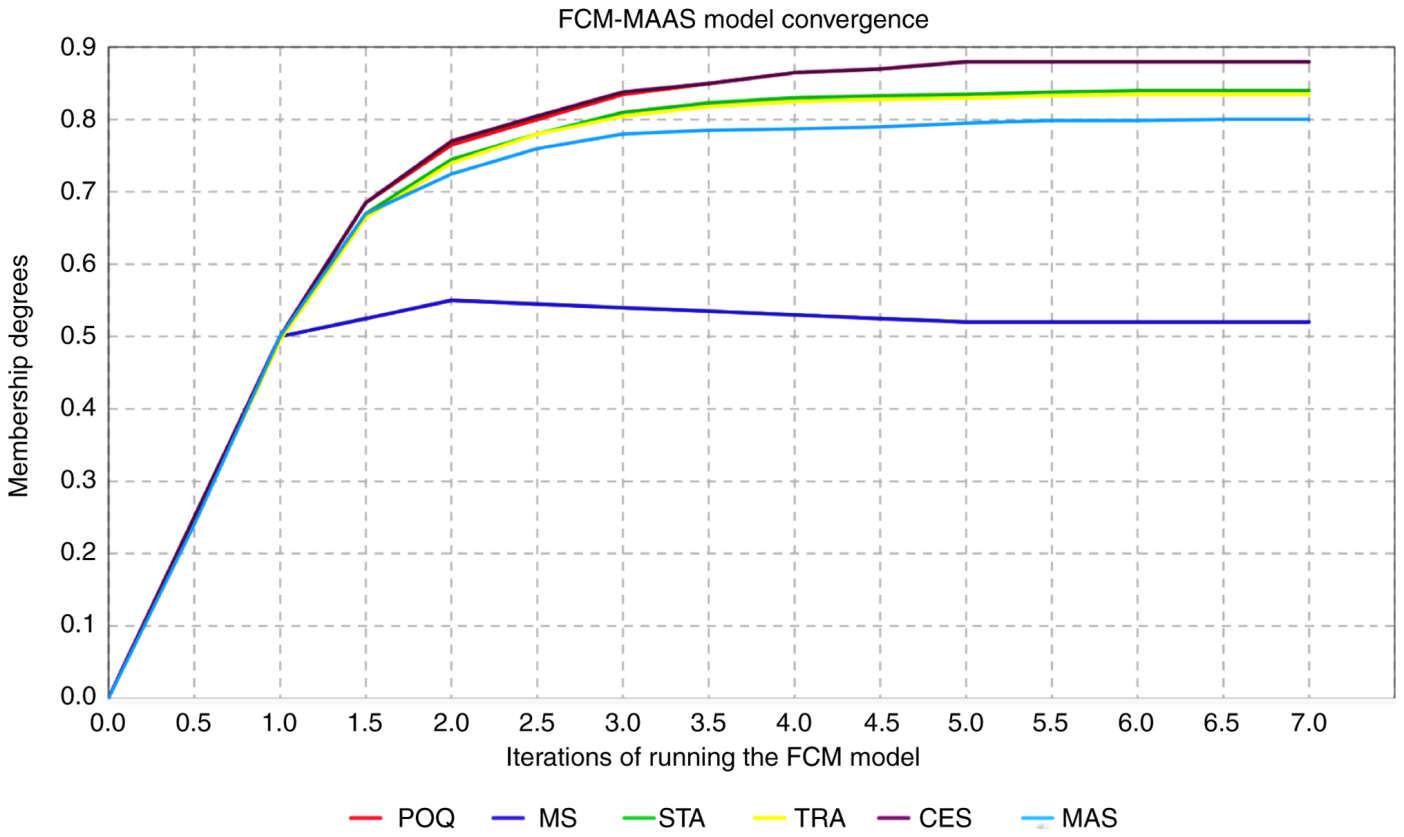
Convergence of the FCM model, illustrating the predicted changes in maternal antenatal attachment (MAAS) based on input variables. The activation vector, derived from fuzzified data, represents the dynamic impact of pregnancy-related psychological factors, including anxiety, marital satisfaction, and depression levels. The model estimates fluctuations over multiple iterations until equilibrium is reached, demonstrating the predictive stability of the proposed approach. FCM, fuzzy cognitive map; MAAS, maternal antenatal attachment scale; CESD, Centre for Epidemiologic Studies Depression Scale; MAAS, STATE ANXIETY, maternal antenatal attachment scale; POQ, pregnancy outcome questionnaire; MS, TRAIT ANXIETY, marital status, marital satisfaction.

The activation vector indicates that woman-1 experienced a slight increase in anxiety about the pregnancy outcome (POQ=0.357) and a strong improvement in her marital satisfaction (MS=0.729), etc. The output variable MAAS-total is set to 0, as an initial value of its fluctuation. By applying formula (7), the multiplication (MAAS-total-Activation Vector) x (MAAS-total) returns the model's estimation of MAAS fluctuations from (0) for pregnant woman-1. The model estimates the of MAAS-total change is (FCM-MAAS=0,70024), indicating that woman-1 is expected to experience a strong increase in prenatal attachment. The model reaches equilibrium and converges, as shown in Fig. 2.

The model's estimation approximates the actual MAAS-total value in the sample data (0.723) for woman-1. To examine the FCM predictive value, the t-test was applied for all the women in the sample, resulting in P-values >0.05, thus indicating that there was no statistically significant difference between the model estimations and the actual sample data. The present study analyzed all 9 cases similarly. The causal combination for each case with its corresponding consistency, coverage and validation is presented in Table V.

## Discussion

The intricate and multidimensional nature of the bond between a mother and her developing fetus has been extensively studied in maternal and child health research (44-47). A key aspect of this prenatal relationship is maternal-fetal attachment, which is described in scientific literature as the distinctive connection that forms between a pregnant woman and her unborn child (48-52). This attachment is considered a fundamental component of the mother-child relationship during pregnancy.

The present study investigated the predictors of elevated MAAS levels in pregnant women before delivery. The results indicated that, for pregnancies following natural conception, women with a low POQ score, a favorable marital status (marital satisfaction), and low levels of both state and trait anxiety, as well as low depressive symptoms (CESD), tended to have higher scores on the MAAS.

The present study explored the determinants of heightened MAAS scores among expectant mothers during the antepartum period and the findings reveal that in naturally conceived pregnancies, several factors correlate with elevated MAAS scores. These include lower POQ results, positive marital satisfaction, reduced state and trait anxiety levels, and minimal depressive symptomatology as measured by the CESD. The present study examined the predictive factors associated with enhanced maternal-fetal bonding in the prenatal period. The research identified multiple variables linked to higher MAAS scores, which indicate the emotional connection between a pregnant woman and her unborn child: Lower pregnancy-related stress (measured by a low POQ score), a happy marriage or relationship, low levels of general (trait) and situational (state) anxiety, and fewer depression symptoms.

These findings apply to women who conceived naturally, without fertility treatments. The present study indicated that pregnant women who experienced lower levels of stress, maintain positive interpersonal relationships, and exhibit reduced symptoms of anxiety and depression are more likely to develop stronger emotional attachments to their fetuses.

By utilizing this information, key maternal healthcare providers, such as obstetricians and midwives, can simplify the assessment process by concentrating on essential elements, thus reducing the number of medical examinations, associated costs, and the complexity of data required for evaluations. For pregnancies achieved through ART, the present study found that the POQ score was not necessary for predicting MAAS levels, simplifying the assessment process in these cases. In ART pregnancies, marital satisfaction, combined with low anxiety and depressive symptoms, serve as sufficient predictors of high MAAS scores before childbirth (53,54).

The present study examined several variables influencing POQ scores before delivery in both natural and ART pregnancies. The key findings included POQ scores that were positively associated with a low income, a favorable marital status and a low state of anxiety. These factors are critical for predicting high POQ levels before birth. For non-assisted births, income is not a significant factor in assessing POQ. For ART pregnancies, high POQ scores are associated with women who have a low income, high maternal antenatal attachment intensity (MAAS-Intensity), a favorable marital status and a low level of anxiety. Key factors for assessing POQ in ART cases also include a low income, an advanced gestational age and a low level of anxiety, distinguishing these assessments from those for spontaneous conception.

High levels of depressive symptoms, as indicated by the CESD, were found to be more prevalent in younger women who experienced marital satisfaction and high levels of state anxiety. In cases of natural conception, women with low marital satisfaction, a low level of anxiety and high POQ scores are predicted to exhibit high CESD levels (55). In assessing pregnancies resulting from ART, it is sufficient to evaluate the predictive power of the POQ alone to anticipate high levels of CESD. This highlights the unique predictive capabilities of POQ in these cases. Overall, the present study emphasizes the significance of marital satisfaction, state anxiety, and specific socioeconomic factors in predicting pregnancy outcomes (56-58). It also highlights distinct considerations for ART pregnancies compared to those resulting from natural conception (52).

The present study pinpoints the specific sets of factors (causal combinations) that are both necessary and sufficient to predict heightened levels of maternal antenatal attachment (MAAS), positive pregnancy outcomes (POQ) and diminished levels of depression (CESD). These factors encompass marital status, state and trait anxiety, income, and the week of pregnancy. The results of the present study indicate that distinctive conditions apply based on whether the pregnancy was natural or assisted (55,56).

The study on maternal antenatal attachment, pregnancy outcomes and depression, using FSQCA and FCMs present several advantages, constraints and potential areas for future research. The innovative methodology employed in the study is a notable strength. The findings extend beyond group-level insights to individual case assessments through the FCM model. FCMs allow clinicians to model dynamic interactions between key psychological and social factors for personalized scenario analysis. By adjusting input variables, such as marital satisfaction, trait anxiety, or social support, clinicians can simulate changes in the psychological well-being of a patient, facilitating targeted interventions.

This adaptability renders FCM a valuable tool for individualized care, predicting patient-specific outcomes based on real-time adjustments. Future integration of FCM models into clinical decision-support tools could enhance their utility, enabling healthcare providers to refine treatment plans according to each patient's unique profile. This personalized approach highlights the relevance of these findings in guiding tailored mental health interventions for pregnant women.

The application of FSQCA and FCM facilitates the analysis of intricate and non-linear relationships among various factors, which conventional regression techniques may not adequately capture. The comprehensive analysis successfully identifies the necessary and sufficient combinations of factors that can predict different pregnancy-related outcomes. This can lead to more tailored and effective interventions for pregnant women. Additionally, the findings offer practical utility, providing actionable insight that maternity care professionals, such as midwives and obstetricians can use to better predict and manage the conditions of pregnant women, ultimately enhancing prenatal care and outcomes.

The limitations of the present study may include the sample size and diversity. Insufficient diversity or an inadequate sample size in a study population may result in outcomes that lack generalizability or applicability to broader populations, which is a crucial consideration for determining clinical relevance in practical settings (58). Although larger sample sizes can provide additional power and reveal subtle patterns, the sample size in the present study was determined based on power calculations to adequately address our research questions. Resource constraints and logistics limited the ability of the authors to expand the sample size without compromising study quality and feasibility.

The present study explored the interactions between psychosocial, demographic and economic factors that affect maternal psychological well-being during pregnancy. Given the exploratory and predictive nature of the present study, a control group was excluded. Psychological well-being during pregnancy is influenced by multiple interacting factors, making it difficult to define a universally applicable ‘control’ condition. The aim of the present study was to identify patterns within a naturally occurring population, rather than comparing treated vs. untreated groups. The present study aimed to reflect real-life clinical scenarios in which pregnant women experience varying degrees of anxiety, depression and attachment to their fetus. These findings apply to a broader population of pregnant women, enhancing their relevance for obstetricians and midwives in clinical practice. The methodologies employed, particularly FSQCA and FCMs, did not require a control group to identify meaningful patterns. FSQCA identifies the necessary and sufficient conditions across cases. FCM models the dynamic interplay of factors within a population, making it well-suited for assessing individual differences without a control group (59).

Ethical and practical constraints also exist. Recruiting a control group while ensuring meaningful comparisons poses ethical and logistical challenges. Additional screening was required, potentially excluding participants with subclinical psychological distress and introducing bias to the study.

Additionally, the use of FCM relies heavily on expert opinions, which can introduce subjectivity into the model, potentially affecting the reproducibility and objectivity of the findings. Furthermore, the application of findings obtained through FSQCA can be complex, particularly for practitioners unfamiliar with this technique, potentially impeding the interpretation of the results.

Despite these limitations however, both FCM and FSQCA provide valuable insight into complex systems and causal relationships that traditional quantitative methods may overlook. To address the potential biases in FCM, researchers can employ multiple experts and implement rigorous validation processes to enhance the reliability of the model. Similarly, for FSQCA, providing comprehensive documentation and conducting workshops or training can help stakeholders to better understand and apply sessions the results, ultimately improving the practical impact of the research. Overall, the present study greatly contributes to the comprehension of intricate interactions among various factors that influence pregnancy-related results and introduces an innovative technique that effectively captures these interactions in a clinically beneficial manner (60-62).

Future studies may consider expanding the sample size and recruiting a more diverse group of participants to enhance the generalizability of the results. Conducting longitudinal research may afford a deeper appreciation of how the associations between these variables develop and alter over time, and how these dynamics ultimately influence both maternal and child well-being in the long run. A cross-cultural research design can explore these associations in various cultural contexts and may provide insight into how cultural factors influence prenatal and mental health outcomes.

Comparisons with alternative models would be beneficial in assessing the effectiveness of FSQCA and FCM in relation to other predictive models, as this could either validate or challenge their superiority in managing complex clinical data. Lastly, incorporating additional variables may prove advantageous. Future research could explore additional factors, such as psychological resilience, social support, or specific health behaviors, to assess their impact on the primary outcomes. Further investigations are encouraged to determine how these predictive models perform across different demographic and clinical subgroups.

In conclusion, to the best of our knowledge, the present study is the first to utilize the FSQCA and FCM in identifying and assessing the alternative causal paths that lead to high levels of anxiety regarding pregnancy outcomes, maternal prenatal attachment to the unborn child, and depressive symptoms before birth. The present study demonstrates the applicability of FSQCA and FCM. The models produced achieved high consistency and acceptable coverage in each case and exhibited predictive value.

The prevalence of antenatal depression may vary significantly across countries; moreover, anxiety regarding pregnancy outcomes and antenatal depression are often associated with cognitive problems and psychopathology in offspring. Thus, it is essential to utilize fuzzy logic, incorporate expert judgment and intuition, and analyze alternative causal paths, in order to make decisions concerning the best course of action for each pregnant woman. The results of the present study suggest that further research is required to focus on testing the approach with larger data sets and improving the coverage of the models.

## Figures and Tables

**Table I:** TFN of the concepts used in the FSQCA.

Variable in the FSQCA analysis	Triangular fuzzy numbers (TFNs) (a, m, b) for each variable
POQ-pregnancy outcome	1, 4, 4
CESD values	0, 3, 3
MAAS total	1, 5, 5
MAAS QUALITY-maternal antenatal attachment scale	1, 5, 5
MAAS INTENSITY-maternal antenatal attachment scale	1, 5. 5
Marriage satisfaction before birth (MS)	1, 10, 10
STATE_ANXIETY	1, 4, 4
TRAIT_ANXIETY	1, 4, 4
Week of pregnancy	20, 40, 40
Income	1, 6, 6
Age	17, 52, 52
Education	1, 6, 6

POQ, pregnancy outcome questionnaire; CESD, Center for Epidemiologic Studies Depression Scale; MAAS, maternal antenatal attachment scale; MS, marital satisfaction before birth; TFN, triangular fuzzy number; FSQCA, fuzzy-set qualitative comparative analysis.

**Table II:** A total of 9 cases were analyzed: MAAS, POQ and CESD for NC and ART pregnancies.

Output variable	Sample	Input variables
High MAAS (total)	135 Pregnant women (total sample)	Age, income, education, week of pregnancy, POQ, MS, state anxiety, trait anxiety, CESD
	115 Non-assisted pregnancy cases	Age, income, education, week of pregnancy, POQ, MS, state anxiety, trait anxiety, CESD
	20 Assisted pregnancy cases	Age, income, education, week of pregnancy, POQ, MS, state anxiety, trait anxiety, CESD
High POQ	135 Pregnant women (total sample)	Age, income, education, week of pregnancy, MAAS quality, MASS intensity, MS, state anxiety, trait anxiety, CESD
	115 Non-assisted pregnancy cases	Age, income, education, week of pregnancy, MAAS quality, MASS intensity, MS, state anxiety, trait anxiety, CESD
	20 Assisted pregnancy cases	Age, income, education, week of pregnancy, MAAS quality, MASS intensity, MS, state anxiety, trait anxiety, CESD
High CESD	135 Pregnant women (total sample)	Age, income, education, week of pregnancy, MAAS quality, MASS intensity, MS, state anxiety, trait anxiety, POQ
	115 Non-assisted pregnancy cases	Age, income, education, week of pregnancy, MAAS quality, MASS intensity, MS, state anxiety, trait anxiety, POQ
	20 Assisted pregnancy cases	Age, income, education, week of pregnancy, MAAS quality, MASS intensity, MS, state anxiety, trait anxiety, POQ

MAAS, maternal antenatal attachment scale; POQ, pregnancy outcome questionnaire; CESD, Center for Epidemiologic Studies Depression Scale; NC, natural conception; ART, assisted reproductive technology.

**Table III:** Selected causal combinations that lead to high levels of MAAS total before birth.

Causal combination	Age	Education	Income	Week of Pregnancy	POQ	MS	STATE ANXIETY	TRAIT ANXIETY	CESD values
8	0	0	0	0	0	1	0	0	0
40	0	0	1	0	1	1	0	0	0
72	0	0	1	1	0	1	0	0	0
104	0	0	1	1	1	1	0	0	0
136	0	1	0	1	0	1	0	0	0
168	0	1	1	1	0	1	0	0	0
200	0	1	1	1	1	1	0	0	0
232	0	1	1	0	0	1	0	0	0
264	1	0	0	1	0	1	0	0	0
296	1	0	0	1	1	1	0	0	0
328	1	0	1	1	0	1	0	0	0
360	1	0	1	1	1	1	0	0	0
392	1	1	0	1	0	1	0	0	0
424	1	1	1	0	0	1	0	0	0
456	1	1	1	0	1	1	0	0	0

The number 0 indicates low i.e., not high values in the corresponding variables, while the number 1 indicates high variables values. For causal combination the number 8, age=0 indicates a woman of a young age. Similarly, the zeros (0) indicate low education, low income, early weeks of pregnancy, low values n POQ, State-Anxiety, Trait-Anxiety, and CESD. The MS=1 indicates a woman that enjoys a high level of marital satisfaction. MAAS, maternal antenatal attachment scale; POQ, pregnancy outcome questionnaire; MS, marital satisfaction before birth; CESD, Center for Epidemiologic Studies Depression Scale.

**Table IV:** The MAAS-total activation vector of woman-1 data.

Activation vector	POQ	MS	STATE	TRAIT	CESD	MAAS-total
Woman-1	0.357	0.729	0.083	0.267	0.117	0

MAAS, maternal antenatal attachment scale; POQ, pregnancy outcome questionnaire; MS, marital satisfaction before birth; CESD, Center for Epidemiologic Studies Depression Scale.

**Table V:** The resulting causal combinations for each of the 9 cases.

No.	Cases	Sample	Cause-effect set of rules causal combination	Consistency	Coverage	Validation (t-test/P-value)
1	Causal combinations for assessing MAAS total before birth	135	IF (not POQ) AND (MS) AND (not STATE ANXIETY) AND (not TRAIT ANXIETY) AND (not CESD) THEN <High Level of	>0.9 MAAS before birth>	>0.4	>0.05
2	Causal combinations for assessing MAAS total before birth for NC pregnancies	115	IF (not POQ) AND (MS) AND (not STATE ANXIETY) AND (not TRAIT ANXIETY) AND (not CESD) THEN <High Level of MAAS before birth for NC pregnancies>	>0.9	>0.4	>0.05
3	causal combinations for assessing MAAS total before birth for ART pregnancies	20	IF (MS) AND (not STATE ANXIETY) AND (not TRAIT ANXIETY) AND (not CESD) THEN <High Level of MAAS before birth for ART pregnancies>	>0.9	>0.43	>0.05
4	Causal combinations for assessing POQ before birth	135	IF (not INCOME) AND (MS) AND (not STATE ANXIETY) THEN <High Level of POQ before birth>	>0.9	>0.49	>0.05
5	Causal combinations for assessing POQ before birth for NC pregnancies	115	IF (MS) AND (not STATE ANXIETY) THEN <High Level of POQ before birth for NC pregnancies>	>0.9	>0.48	>0.05
6	Causal combinations for assessing POQ before birth for ART pregnancies	20	IF (not INCOME) AND (WEEK of Pregnancy) AND (MAAS-Int) AND (MS) AND (not TRAIT ANXIETY) THEN <High Level of POQ before birth for ART	>0.9 pregnancies>	>0.48	>0.05
7	Causal combinations for assessing CESD before birth	135	IF (not AGE) AND (not MS) AND (STATE ANXIETY) THEN <High Level of CESD before birth>	>0.9	>0.48	>0.05
8	Causal combinations for assessing CESD before birth for NC pregnancies	115	IF (not MS) AND (STATE ANXIETY) AND (POQ) THEN <High Level of CESD before birth for NC pregnancies>	>0.9	>0.5	>0.05
9	Causal combinations for assessing CESD before birth for ART pregnancies	20	IF (POQ) THEN <High Level of CESD before birth for ART pregnancies>	>0.9	>0.86	>0.05

MAAS, maternal antenatal attachment scale (total); POQ, pregnancy outcome questionnaire; MS, marital satisfaction before birth; CESD, Center for Epidemiologic Studies Depression Scale; NC, natural conception; ART, assisted reproductive technology.

## Data Availability

The data generated in the present study may be requested from the corresponding author.
